# Effects of adjuvant huaier granule therapy on survival rate of patients with hepatocellular carcinoma

**DOI:** 10.3389/fphar.2023.1163304

**Published:** 2023-05-11

**Authors:** Ke Shi, Yufei Bi, Xuanwei Zeng, Xianbo Wang

**Affiliations:** Center of Integrative Medicine, Beijing Ditan Hospital, Capital Medical University, Beijing, China

**Keywords:** traditional Chinese medicine, huaier granule, complementary alternative medicine, hepatocarcinoma carcinoma, prognosis

## Abstract

**Objective:** Clinical trials have reported that Huaier granule inhibits the recurrence of hepatocellular carcinoma (HCC) after resection. However, its efficacy in patients at different clinical stages of HCC remains unknown. We investigated the effects of Huaier granule on the 3-year overall survival (OS) rate of patients at different clinical stages.

**Design:** This cohort study included 826 patients with HCC, screened between January 2015 and December 2019. The patients were divided into Huaier (n = 174) and control groups (n = 652), and the 3-year OS rates were compared between the two groups. To eliminate bias caused by confounding factors, propensity score matching (PSM) was performed. We used the Kaplan-Meier method to estimate OS rate and tested the difference using the log-rank test.

**Results:** Multivariable regression analysis revealed that Huaier therapy was an independent protective factor for 3-year survival rate. After PSM (1:2), the Huaier and control groups comprised 170 and 340 patients, respectively. The 3-year OS rate was remarkably higher in the Huaier group than in the control group (adjusted hazard ratio [aHR]: 0.36; 95% confidence interval: 0.26–0.49; *p* < 0.001). The aHR for Huaier use for 3–12, 12–24, and >24 months was 0.48, 0.23, and 0.16, respectively, indicating a dose-response pattern. For the 3–12-, 12–24-, and >24-month groups, the 3-year OS rate was 54.1%, 68.6%, and 90.4%, respectively. Multivariate stratified analysis confirmed that the mortality risk in Huaier users was lower than that in non-Huaier users in most subgroups.

**Conclusion:** Adjuvant Huaier therapy improved the OS rate in patients with HCC. However, these findings require further verification through prospective clinical studies.

## Introduction

Primary liver cancer is the fourth most common cause of cancer-related deaths worldwide, with increasing annual incidence and poor prognosis (Arnold et al., 2020). Hepatocellular carcinoma (HCC) accounts for 85–90% of primary liver cancers, with 782,000 deaths annually (Bray et al., 2018). Liver transplantation, surgical resection, transarterial chemoembolization, and radiofrequency ablation are the most widely used primary treatment methods for patients with early-stage HCC (Yang et al., 2019). However, owing to unobvious symptoms at an early stage, most patients are diagnosed at the middle and advanced stages, with a 5-year survival rate of approximately 18% (Jemal et al., 2017). Hence, the high mortality rate of HCC patients poses a challenge for effective treatment, and more effective therapies are urgently needed.

Traditional Chinese medicine (TCM) is commonly used to treat HCC (Li, 2020; Li Y. et al., 2022). Recently, most Asian patients with HCC have begun to use TCM as an alternative or a complementary treatment. TCM improves clinical symptoms and the quality of life of patients with HCC, enhancing the median survival time and survival rate (Liu et al., 2019; Changou et al., 2021; Li K. et al., 2022). Previous studies have reported that Huaier granule exerts antitumor effects by inhibiting cell proliferation, promoting apoptosis, enhancing autophagy, inhibiting angiogenesis, and modulating immune response (Zhang et al., 2022; Wu et al., 2014; Wang et al., 2012). Moreover, clinical studies have elucidated the effect of Huaier granule on recurrence and overall survival (OS) rates after curative resection of HCC (Chen et al., 2018; Luo and Hu, 2023). However, most studies have only observed patients with early-stage HCC after curative resection. The effects of adjuvant Huaier granule therapy on the OS of patients with multiple clinical stages remains unknown. Considering the widespread use of Huaier granules and the high mortality rate of HCC patients (Li Y. et al., 2022; Li K. et al., 2022), the connection between Huaier granules and prognosis should be investigated.

In this study, we evaluated the efficacy of Huaier granule in the treatment of patients at different clinical stages and provide evidence for success of adjuvant therapy in these patients.

## Materials and methods

### Study populations

**FIGURE 1:**
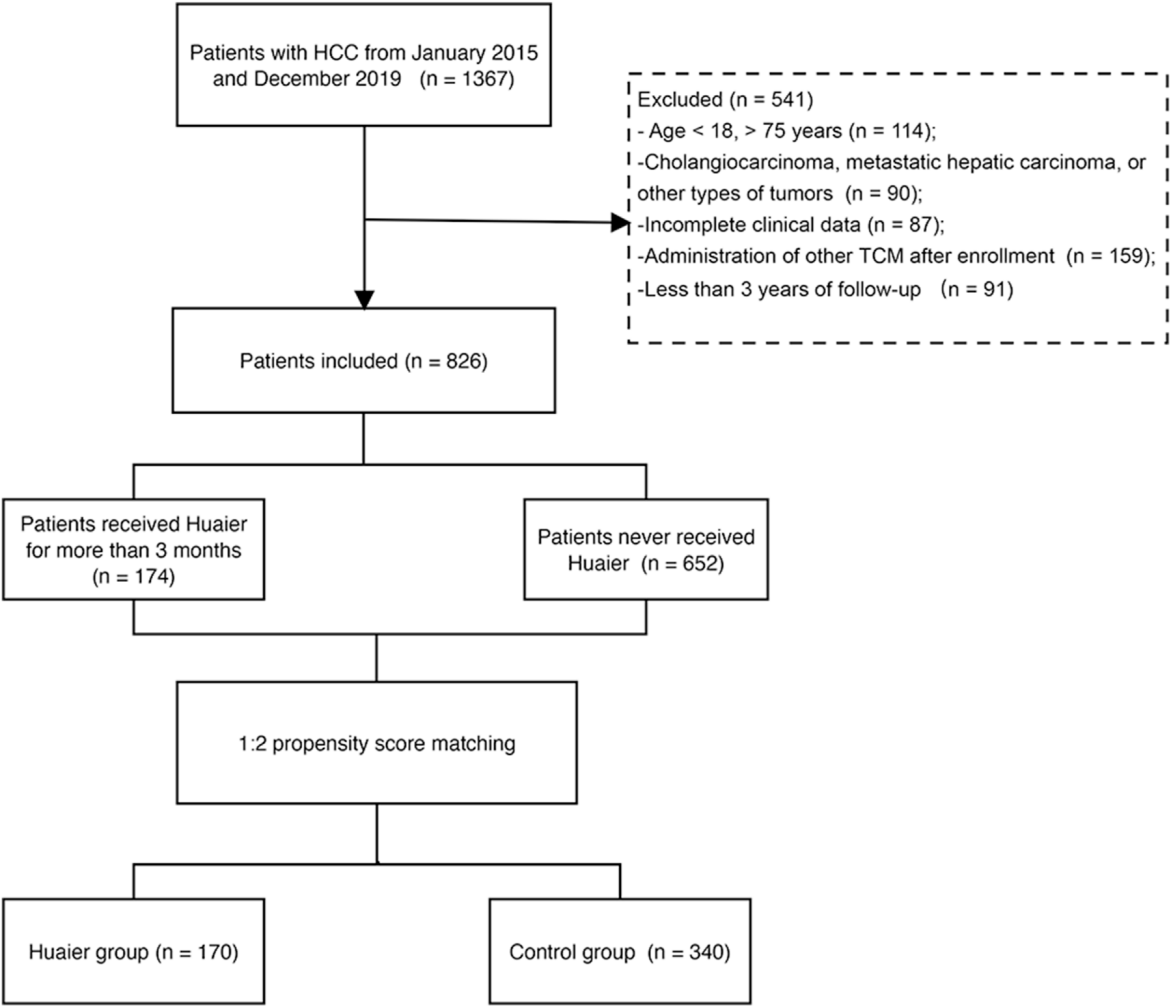
Flowchart of the enrollment of patients with hepatocellular carcinoma.

We screened 1,367 patients with HCC between January 2015 and December 2019 at the Beijing Ditan Hospital, Capital Medical University. HCC diagnosis was based on histological or radiological analysis, including computed tomography (CT) or magnetic resonance imaging (MRI), and was assessed by clinically experienced physicians (Omata et al., 2017). Individuals between 18 and 75 years of age who were diagnosed with HCC were included in this study. The exclusion criteria were as follows: 1) age <18, or >75 years; 2) cholangiocarcinoma, metastatic hepatic carcinoma, or other types of tumors; 3) incomplete clinical data; 4) administration of other TCM after enrollment; and 5) less than 3 years of follow up. Finally, 826 patients were included in this study (Figure 1).

The patients were divided into Huaier and control groups according to the cumulative use of Huaier granule for >3 months during the follow-up period. Huaier granules, the aqueous product of Huaier extract, has been approved by the State Food and Drug Administration (SFDA) of China (No. Z20000109) and is used to treat liver, lung, gastric, and breast cancers (Chen et al., 2018). In this study, each Huaier granule packet contained 20 g Huaier extract, which was taken orally with 100 mL of water three times daily. The control group was treated with local surgical resection, minimally invasive treatments, including transarterial chemoembolization, radio frequency ablation, microwave ablation, and selective internal radiation therapy; and palliative treatment, including palliative symptomatic treatment, systemic chemotherapy, sorafenib, and lenvatinib (European Association for the Study of the Liver, 2018). The baseline data included the time of HCC diagnosis at our hospital. The primary endpoint was patient death within 3 years or the end of the 3-year follow-up period, whichever came first. The study was performed in accordance with the ethical guidelines of the Declaration of Helsinki (World Medical Association, 2023) and was approved by the ethics committee of the Beijing Ditan Hospital.

### Clinical data collection

Demographic characteristics and baseline data, including age, sex, history of HCC in family members, diabetes, hypertension, platelet count (PLT), prothrombin time (PT), international normalized ratio (INR), and levels of alanine aminotransferase (ALT), aspartate aminotransferase (AST), total bilirubin (TBIL), γ-glutamyl transferase (γ-GGT), serum albumin (ALB), serum creatinine (Cr), and alpha-fetoprotein (AFP), were recorded from a computerized database during the 24 h of enrollment. Moreover, tumor characteristics, such as tumor number, size, vascular invasion, and metastasis, were recorded based on the imaging data at baseline. The model for end-stage liver disease (MELD) and Child-Turcotte-Pugh (CTP) scores were used to estimate the severity of liver disease (Botero and Lucey, 2003; Wiesner et al., 2003). Routine laboratory tests and radiological examinations were performed every 3 months.

### Statistical analysis

SPSS (version 25.0; SPSS, Inc., Chicago, IL, USA) and R software (version 4.1.2; R Foundation, Vienna, Austria) were used for statistical analysis of data. Continuous variables are expressed as mean ± standard deviation (SD) or median with interquartile range (IQR) and were compared using an independent Student’s *t*-test or the Mann-Whitney test. Categorical variables are expressed as numbers, percentages, and 95% confidence intervals (CI) and were compared using the chi-square test or Fisher’s exact test. Univariate and multivariate Cox proportional hazards regression models were used to determine the effect of Huaier administration on survival rate. Adjusted hazard ratios (aHR) and 95% CIs were calculated.

Propensity score matching (PSM) is used to reduce bias due to potential confounding factors between groups (Rusthoven et al., 2016; Tian et al., 2022). Based on variables related to outcome, a logistic regression model was used to perform PSM to balance the potential confounding factors. The Huaier and control groups were matched randomly by age, sex, MELD score, AFP level, tumor multiplicity, tumor size, and Barcelona Clinic Liver Cancer (BCLC) staging. The nearest neighbor matching algorithm with a caliper width of 0.05 was used to undertake one-to-two matching without replacement. The caliper width was 0.05 times the standard deviation of the logit of the propensity score (Austin, 2011), which eliminated at least 99% of the bias by confounding variables (Rusthoven et al., 2016).

Furthermore, the patients were divided into three subgroups according to the duration of Huaier use: 3–12, 12–24, and >24 months. The Kaplan-Meier method was used to compare survival rates, and the log-rank test was used to compare significant differences. The Cox proportional hazards regression model was used to calculate multivariate aHR and 95% CI of mortality associated with Huaier administration. Furthermore, multivariate stratified analysis was performed as a sensitivity analysis to assess the effect of Huaier therapy in the different subgroups. A forest plot was constructed to compare the hazard ratio (HR) for 3-year mortality rate between the Huaier and the control groups. All tests were two-tailed, and a *p*-value <0.05 was considered statistically significant.

## Results

### Baseline characteristics

In this study, 174 out of the 826 patients received Huaier granule for >3 months, whereas 652 patients did not receive Huaier granule. During the 3-year follow-up period, 63 Huaier users and 465 non-Huaier users died. The baseline characteristics of the unmatched and matched cohorts are presented in Table 1 and Table 2, respectively. The median age of the patients was 57.0 years (IQR: 50.0–63.0), with male predominance (n = 639, 77.3%). Most patients (51.8%) received minimally invasive treatment, 342 (41.4%) received palliative treatment, and 56 (6.8%) underwent surgical resection. Before PSM, in the control group, the proportion of participants with tumor size ≥5 cm, multiple tumors, and AFP ≥400 ng/mL was higher than that in the Huaier group; moreover, the control-group patients were older and had higher MELD score, INR, PLT, and levels of ALT, AST, TBIL, γ-GGT, and Cr than those in the Huaier group (all *p* < 0.001). In the control group, a significant percentage of patients were in BCLC stages B–D (*p* < 0.001). After PSM, the demographic and laboratory data were consistent between the two groups, and a matched cohort of 510 patients was included in the analysis. Among them, 170 and 340 patients belonged to the Huaier and control groups, respectively.

**TABLE 1:** Demographic and clinical characteristics of patients with hepatocellular carcinoma.

	All patients (n = 826)	Huaier group (n = 174)	Control group (n = 652)	*p*-Value
**Patients background**				
Age, y	57.0 (50.0–63.0)	54.0 (47.0–60.0)	57.0 (50.0–63.0)	< 0.001
Sex, male	639 (77.3)	140 (80.4)	499 (76.5)	0.216
Family history of HCC	72 (8.7)	21 (12.1)	51 (7.8)	0.078
Smoking	342 (41.4)	65 (37.4)	277 (42.5)	0.222
Alcohol consumption	332 (40.2)	65 (37.4)	267 (40.9)	0.390
Hypertension	212 (25.6)	42 (24.1)	170 (26.1)	0.604
Diabetes	180 (21.8)	36 (20.7)	144 (22.1)	0.692
Cirrhosis	738 (89.3)	151 (86.8)	587 (90.0)	0.217
**Etiology**				0.062
HBV	580 (70.2)	135 (77.6)	445 (68.3)	
HCV	103 (12.5)	15 (8.6)	88 (13.5)	
Other	143 (17.3)	24 (13.8)	119 (18.3)	
**Laboratory parameters**				
MELD score	8.8 (5.4–12.8)	7.2 (4.2–10.3)	9.4 (5.7–14.1)	< 0.001
ALT, U/L	36.8 (23.1–62.4)	31.3 (22.0–46.4)	39.0 (23.8–68.0)	0.001
AST, U/L	52.1 (30.5–113.3)	34.4 (25.4–55.5)	59.3 (33.6–132.0)	< 0.001
TBIL, µmol/L	25.8 (14.4–48.4)	18.2 (11.5–31.8)	28.9 (16.2–56.2)	< 0.001
ALB, g/L	34.0 ± 6.9	36.8 ± 6.7	33.3 ± 6.8	< 0.001
γ–GGT, U/L	66.8 (30.2–159.9)	40.8 (23.4–95.9)	82.0 (33.3–180.4)	< 0.001
PLT, 10^9^/L	99.1 (65.0–150.1)	88.5 (52.3–134.9)	103.0 (67.4–154.6)	< 0.001
INR	1.2 (1.1–1.3)	1.1 (1.0–1.2)	1.2 (1.1–1.4)	< 0.001
Cr, µmol/L	67.0 (57.0–81.6)	66.6 (57.0–77.0)	68.0 (59.0–84.0)	< 0.001
AFP, ng/ml (≥400)	244 (29.5)	31 (17.8)	213 (32.7)	< 0.001
**Tumor, related indicators**				
Tumor multiplicity (multiple)	412 (49.9)	54 (31.0)	358 (54.9)	< 0.001
Tumor size, cm (≥5)	345 (41.7)	47 (27.0)	298 (45.7)	< 0.001
**BCLC stage**				
0–A	285 (34.5)	95 (54.6)	190 (29.1)	< 0.001
B	252 (30.5)	44 (25.3)	208 (31.9)	< 0.001
C	176 (21.3)	19 (10.9)	157 (24.1)	< 0.001
D	113 (13.7)	16 (9.2)	97 (14.9)	< 0.001
**Types of treatment**				
Resection	56 (6.8)	18 (10.3)	48 (7.4)	< 0.001
Minimally invasive	428 (51.8)	126 (72.4)	312 (47.9)	< 0.001
Palliative	342 (41.4)	30 (17.3)	292 (44.7)	< 0.001

Abbreviations: HBV, hepatitis B virus; HCV, hepatitis C virus; MELD, Model for End-Stage Liver Disease; ALT, alanine aminotransferase; AST, aspartate aminotransferase; TBIL, total bilirubin; ALB, albumin; γ-GGT, γ-glutamyl transferase; Cr, creatinine; INR, international normalized ratio; AFP, alpha-fetoprotein; BCLC, Barcelona Clinic Liver Cancer. Bold values: *p* < 0.05.

**TABLE 2:** Demographic and clinical characteristics of patients with hepatocellular carcinoma after the 1:2 propensity score analysis.

	Huaier group (n = 170)	Control group (n = 340)	*p*-Value
**Patients background**			
Age, y	54.0 (47.0–60.0)	54.0 (48.2–60.0)	0.617
Sex, male	137 (80.6)	266 (78.2)	0.538
Family history of HCC	20 (11.8)	29 (8.5)	0.243
Smoking	63 (37.1)	141 (41.5)	0.338
Alcohol consumption	64 (37.6)	146 (42.9)	0.252
Hypertension	42 (24.7)	78 (22.9)	0.658
Diabetes	36 (21.2)	71 (20.9)	0.939
Cirrhosis	147 (86.5)	307 (90.3)	0.193
**Etiology**			0.293
HBV	132 (77.6)	242 (71.2)	
HCV	15 (8.8)	37 (10.9)	
Other	23 (13.5)	61 (17.9)	
**Laboratory parameters**			
MELD score	7.3 (4.4–10.3)	7.4 (4.2–11.9)	0.507
ALT, U/L	28.5 (19.9–43.9)	28.9 (19.7–39.2)	0.513
AST, U/L	31.3 (21.9–47.0)	34.3 (24.4–49.5)	0.135
TBIL, µmol/L	18.2 (11.8–32.0)	18.6 (11.2–35.4)	0.265
ALB, g/L	36.7 ± 6.7	35.5 ± 6.4	0.162
γ–GGT, U/L	40.8 (23.3–95.9)	51.6 (24.9–101.7)	0.221
PLT, ×10^9^/L	88.5 (51.7–134.9)	92.0 (53.0–142.5)	0.120
INR	1.1 (1.0–1.2)	1.2 (1.0–1.3)	0.459
Cr, µmol/L	66.6 (57.0–77.0)	67.6 (56.0–77.2)	0.687
AFP, ng/ml (≥400)	31 (18.2)	88 (25.9)	0.070
**Tumor–related indicators**			
Tumor multiplicity (multiple)	53 (31.2)	86 (25.3)	0.160
Tumor size, cm (≥5)	47 (27.6)	81 (23.8)	0.348
**BCLC stage**			0.320
0–A	91 (53.5)	173 (50.9)	
B	44 (25.9)	86 (25.3)	
C	19 (11.2)	49 (14.4)	
D	16 (9.4)	32 (9.4)	
**Types of treatment**			0.383
Resection	17 (10.0)	33 (9.7)	
Minimally invasive	123 (72.3)	229 (67.4)	
Palliative	30 (17.6)	78 (22.9)	

Abbreviations: HBV, hepatitis B virus; HCV, hepatitis C virus; MELD, Model for End-Stage Liver Disease; ALT, alanine aminotransferase; AST, aspartate aminotransferase; TBIL, total bilirubin; ALB, albumin; γ-GGT, γ-glutamyl transferase; Cr, creatinine; INR, international normalized ratio; AFP, alpha-fetoprotein; BCLC, barcelona clinic liver cancer.

### Identification of risk factors

According to univariate analysis, age, MELD score, PLT, INR, levels of ALT, AST, TBIL, ALB, γ-GGT, and Cr, AFP ≥400 ng/mL, tumor size ≥5 cm, multiple tumors, and BCLC C–D stages were risk factors for mortality before PSM (all *p* < 0.001), whereas administration of Huaier was identified as a protective factor (HR = 0.32, 95% CI: 0.24–0.41; *p* < 0.001). These significant variables were used for multivariate Cox regression analysis. Finally, MELD score, INR, PLT, levels of TBIL, ALB, and γ-GGT, and PLT, AFP ≥400 ng/mL, multiple tumors, and BCLC C–D stages were established as independent risk factors for 3-year mortality rate (*p* < 0.05 for all factors). Moreover, administration of Huaier was significantly associated with a decreased risk of mortality (aHR = 0.42, 95% CI: 0.32–0.57; *p* = 0.015) (Table 3).

**TABLE 3:** Univariate and multivariate Cox hazards analysis for 3-year overall survival of all patients with hepatocellular carcinoma.

Variables	Univariate analysis		Multivariate analysis	
	HR (95% CI)	*p*-Value	HR (95% CI)	*p*-Value
Huaier treatment	0.32 (0.24–0.41)	**< 0.001**	0.42 (0.32–0.57)	**0.015**
Age, y	1.01 (1.00–1.02)	**< 0.001**		
Gender (male vs. female)	0.91 (0.74–1.11)	0.369		
Cirrhosis (yes vs. no)	1.51 (1.14–2.11)	**0.004**		
Hypertension (yes vs. no)	1.06 (0.99–1.03)	0.118		
Diabetes (yes vs. no)	1.18 (0.76–1.82)	0.456		
MELD score	1.07 (1.06–1.08)	**< 0.001**	1.02 (1.00–1.03)	**0.021**
ALT, IU/L	1.01 (1.01–1.02)	**< 0.001**		
AST, IU/L	1.01 (1.01–1.01)	**< 0.001**		
TBIL, μmol/L	1.04 (1.03–1.05)	**< 0.001**	1.01 (1.00–1.02)	**0.012**
ALB, g/L	0.92 (0.91–0.93)	**< 0.001**	0.96 (0.94–0.98)	**< 0.001**
γ-GGT, IU/L	1.01 (1.01–1.02)	**< 0.001**	1.01 (1.00–1.01)	**0.002**
Platelets, 10^9^/L	1.03 (1.02–1.05)	**< 0.001**	1.03 (1.02–1.04)	**< 0.001**
Cr, µmol/L	1.01 (1.01–1.02)	**< 0.001**		
INR	5.27 (4.29–6.47)	**< 0.001**	2.09 (1.51–2.91)	**< 0.001**
AFP, ng/mL (≥400 vs. < 400)	2.94 (2.46–3.51)	**< 0.001**	1.73 (1.42–2.11)	**< 0.001**
Tumor size, cm (≥5 vs. < 5)	4.22 (3.53–5.04)	**< 0.001**		
Tumor multiplicity (multiple vs. solitary)	2.77 (2.31–3.32)	**< 0.001**	1.51 (1.19–1.96)	**0.001**
BCLC staging (C-D vs. 0-B)	5.21 (4.32–6.26)	**< 0.001**	2.18 (1.75–2.71)	**< 0.001**
Type of treatment				
Resection	0.12 (0.08–0.17)	**< 0.001**	0.50 (0.31–0.78)	**0.003**
Minimally invasive	0.11 (0.09–0.14)	**< 0.001**	0.27 (0.20–0.37)	**< 0.001**
Palliative	References			

Abbreviations: MELD, Model for End-Stage Liver Disease; ALT, alanine aminotransferase; AST, aspartate aminotransferase; TBIL, total bilirubin; ALB, albumin; γ-GGT, γ-glutamyl transferase; Cr, creatinine; INR, international normalized ratio; AFP, alpha-fetoprotein; BCLC, Barcelona Clinic Liver Cancer. Bold values: *p* < 0.05.

### Effect of huaier therapy on survival rate

**FIGURE 2:**
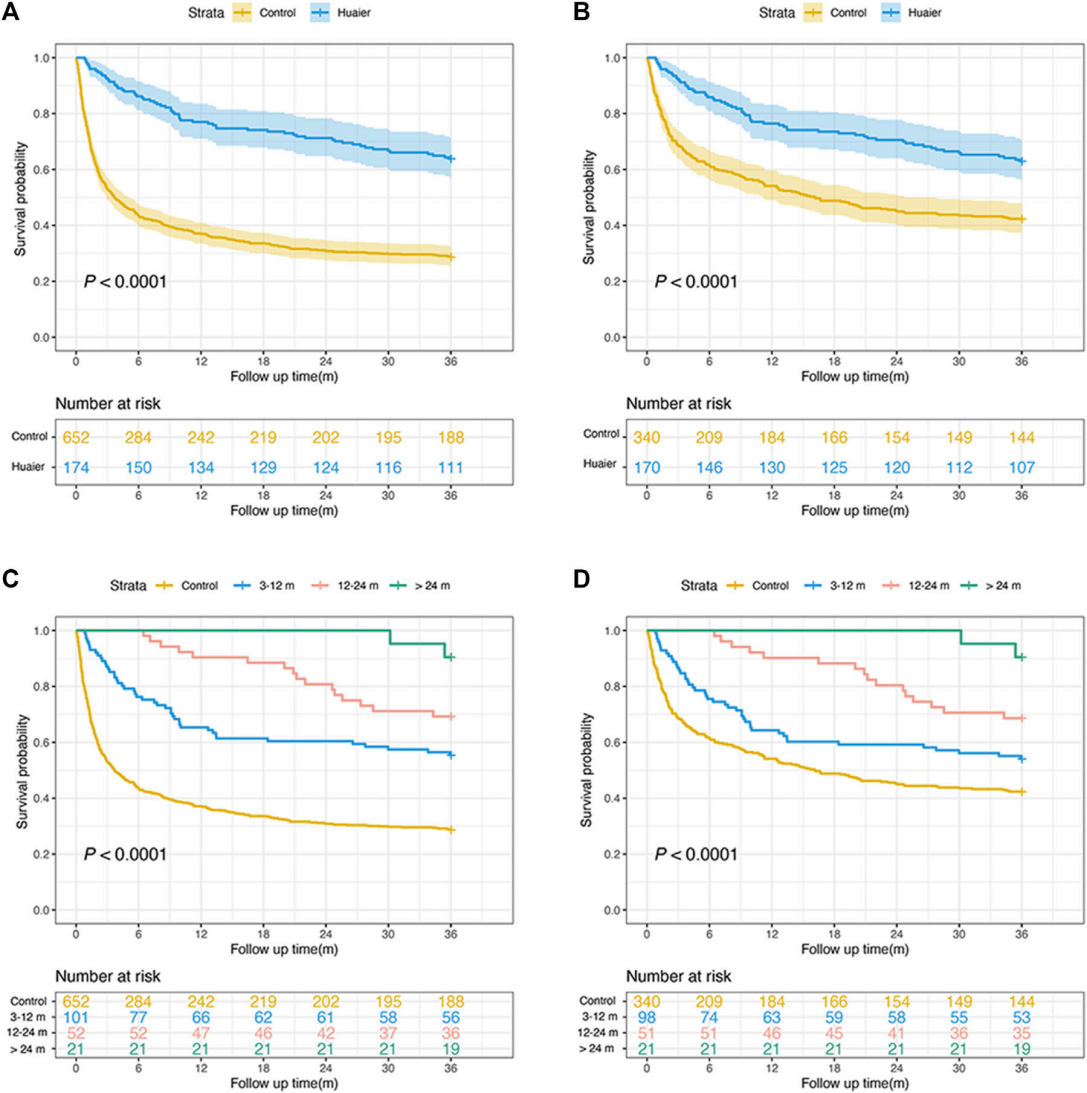
Overall survival rate in patients with hepatocellular carcinoma before **(A)** and after matching with those with and without Huaier use **(B)**. Overall survival rate in patients before **(C)** and after matching **(D)** for different times of Huaier use.

After PSM, the 3-year mortality rate was calculated as 50.8% (259/510 patients). As shown in Figure 2, the Huaier users were significantly associated with an increased OS rate compared to the non-Huaier users, regardless of whether PSM was performed (PSM group: 63.7% vs 28.8%, *p* < 0.0001; non-PSM group: 62.9% vs 42.3%, *p* < 0.0001). Moreover, we divided Huaier users into the following three subgroups according to the duration of Huaier use: 3–12, 12–24, and >24 months. A dose-response relationship between Huaier use and survival probability was observed. Kaplan-Meier analysis revealed that the Huaier and control groups had significantly different OS rates (both before and after PSM, *p* < 0.001).

Similarly, the multivariate Cox proportional hazards model revealed that the adjusted risk of mortality in the Huaier group was significantly lower than in the control group after PSM (aHR = 0.36, 95% CI: 0.26–0.49; *p* < 0.001). Patients using Huaier for 3–12 months (aHR = 0.48, 95% CI: 0.34–0.67; *p* < 0.001), 12–24 months (aHR = 0.23, 95% CI: 0.14–0.40; *p* < 0.001), and >24 months (aHR = 0.16, 95% CI: 0.04–0.64; *p* = 0.010) were at significantly lower risk of mortality (Table 4). Therefore, a longer duration of Huaier administration is associated with a lower mortality rate.

**TABLE 4:** Risk of mortality according to cumulative use of Huaier in unmatched and matched cohorts.

Strata	Total	Death	Crude HR (95%CI)	Adjusted HR (95%CI)
**Unmatched cohort**				
Non-Huaier users	652	465	1 (References)	1 (References)
Huaier users	174	63	0.32 (0.24–0.42) *p* **< 0.001**	0.41 (0.31–0.54) *p* **< 0.001**
3–12 months	101	45	0.42 (0.31–0.57) *p* **< 0.001**	0.50 (0.36–0.69) *p* **< 0.001**
12–24 months	52	16	0.25 (0.15–0.41) *p* **< 0.001**	0.31 (0.18–0.52) *p* **< 0.001**
>24 months	21	2	0.07 (0.02–0.29) *p* **< 0.001**	0.09 (0.01–0.67) *p* = **0.019**
**Matched cohort**				
Non-Huaier users	340	196	1 (References)	1 (References)
Huaier users	170	63	0.49 (0.36–0.65) *p* **< 0.001**	0.36 (0.26–0.49) *p* ***<* 0.001**
3–12 months	98	45	0.66 (0.48–0.92) *p* = **0.014**	0.48 (0.34–0.67) *p* ***<* 0.001**
12–24 months	51	16	0.37 (0.23–0.63) *p* **< 0.001**	0.23 (0.14–0.40) *p* ***<* 0.001**
>24 months	21	2	0.11 (0.03–0.43) *p* = **0.002**	0.16 (0.04–0.64) *p* = **0.010**

Adjusted for age, sex, ALB, γ-GGT, platelets, AFP, MELD, score; BCLC, stage, tumor multiplicity, tumor size, and type of treatment. Abbreviations: ALB, albumin; γ-GGT, γ-glutamyl transferase; AFP, alpha-fetoprotein; MELD, Model for End-Stage Liver Disease; BCLC, barcelona clinic liver cancer; HR, hazard ratio; CI, confidence interval. Bold values: *p* < 0.05.

### Survival analyses by CTP scores, etiology, and tumor characteristics

**FIGURE 3:**
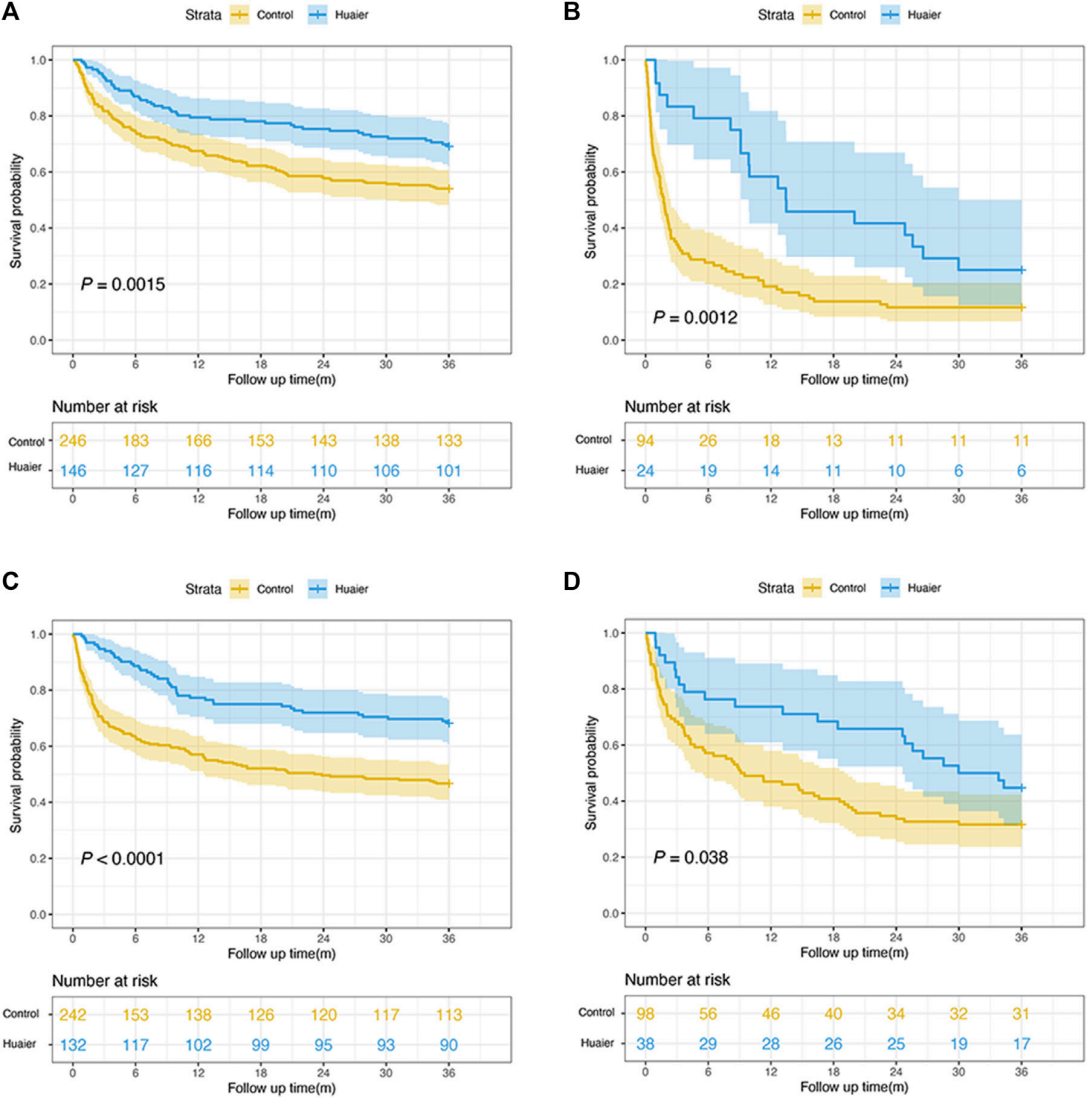
Subgroup analyses of the overall survival rate according to the Child-Pugh classification and etiology. **(A)** Child-Pugh A–B patients; **(B)** Child-Pugh C patients; **(C)** hepatitis B infection patients; **(D)** other etiologies patients.

After PSM, 392 patients belonged to the CTP A–B class and 118 patients were in the CTP C class, and their 3-year OS rates were 69.2% and 25.0% in the Huaier group and 54.1% and 11.7% in the control group, respectively (*p* < 0.005 for both, Figures 3A,B). Most patients (73.3%) had hepatitis B infection and 26.7% had other etiologies. The 3-year OS rates of hepatitis B infection and other etiologies were 68.2% and 44.7% in the Huaier group and 46.7% and 31.6% in the control group, respectively (both *p* < 0.05, Figures 3C,D).

**FIGURE 4:**
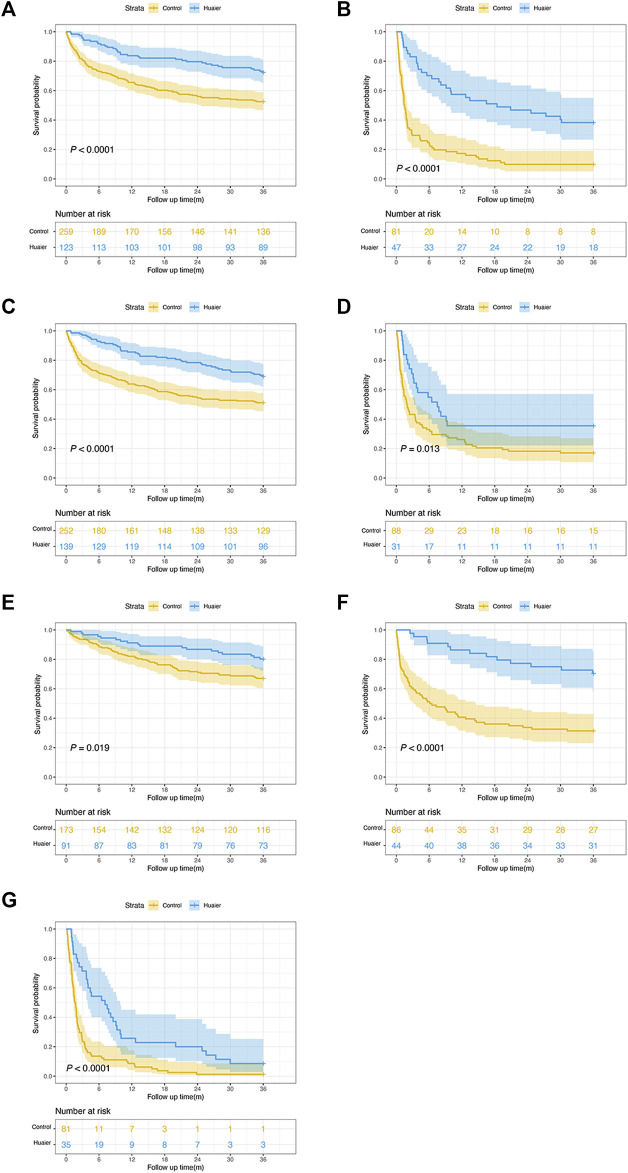
Survival analyses by tumor characteristics for patients with hepatocellular carcinoma. **(A)** tumor size < 5cm; **(B)** tumor size ≥ 5cm; **(C)** AFP level <400 ng/mL; **(D)** AFP level ≥400 ng/mL; **(E)** BCLC stages 0–A; **(F)** BCLC stage B; **(G)** BCLC stages (C–D).

According to tumor characteristics, Kaplan-Meier curves revealed that regardless of tumor size (<5 or ≥5 cm), AFP level (<400 or ≥400 ng/mL), and BCLC stage (0–A, B, C–D), the Huaier users had a significantly higher OS rate than the non-Huaier users (all *p* < 0.05, Figure 4). Therefore, the administration of Huaier granule reduced the risk of mortality in these subgroups.

### Multivariable stratified analysis for huaier therapy

**FIGURE 5:**
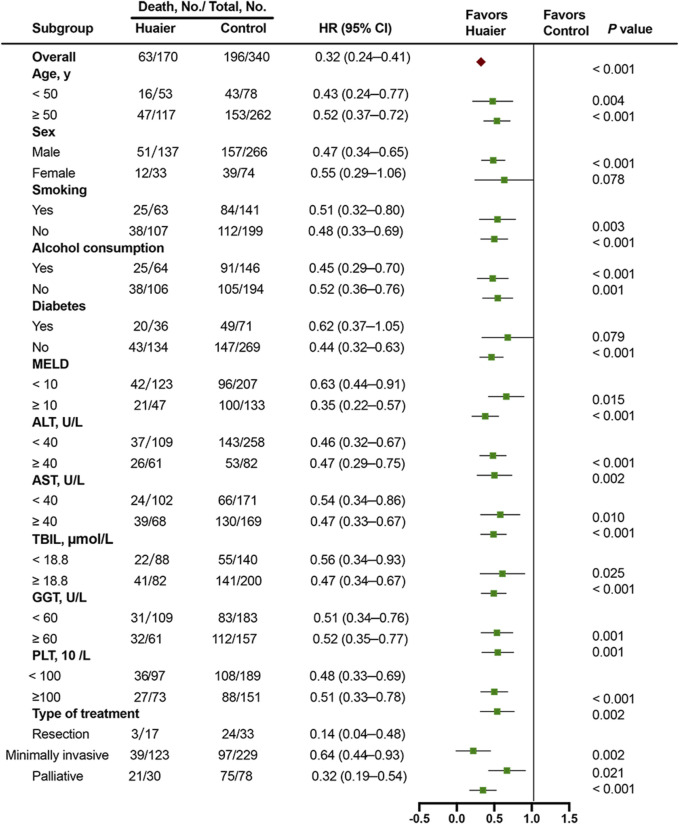
Multivariable stratified analyses of the association between Huaier therapy and survival.

To further investigate the effects of Huaier administration on OS rate, a multivariable stratified analysis was performed for the different subgroups. The effects of Huaier use on the 3-year survival rate of patients in different categories based on age, sex, smoking, alcohol consumption, diabetes, MELD score, levels of ALT, AST, TBIL, γ-GGT, and PLT, and type of treatment were further analyzed (Figure 5). The results indicated that Huaier use was associated with lower mortality risk for the following subgroups of patients: aged ≤50 years, aged ≥50 years, males, smokers and non-smokers, alcohol users and non-users, without diabetes, MELD score ≥10 or <10, ALT ≥40 U/L or <40 U/L, AST ≥40 U/L or <40 U/L, TBIL ≥18.8 μmol/L or <18.8 μmol/L, γ-GGT ≥60 U/L or <60 U/L, PLT ≥100 × 10^9^/L or <100 × 10^9^/L, and those who underwent resection or minimally invasive or palliative treatments (all HR < 1.0). Although the HR was <1.0 for most subgroups, the data were not statistically significant, including female (HR = 0.55, 95% CI: 0.29–1.06) and diabetes (HR = 0.62, 95% CI: 0.37–1.05) subgroups.

## Discussion

HCC is the most common tumor prevalent worldwide, exhibiting rapid progression, worse outcomes, and high mortality rates (Ma et al., 2014). Despite substantial improvements in the treatment regimens for HCC, its prognosis remains poor owing to high recurrence and mortality rates. Therefore, it is important to identify novel therapeutic strategies to improve the survival rate of patients with HCC. TCM has been used in preventing complications and deaths due to HCC (Zhang et al., 2010). Huaier granule, as an adjuvant therapy, inhibits tumor recurrence; however, its role in improving the outcome of patients with HCC at different clinical stages remains unclear. To the best of our knowledge, this is the first clinical study to investigate the association between Huaier therapy and OS rate in patients with HCC at different clinical stages.

Huaier granule exhibits curative effects against several types of cancer, without any therapeutic side effects (Li et al., 2015; Zhang et al., 2019; Zhou et al., 2020). Previous studies have reported that Huaier granule can improve OS rate in patients after curative resection of HCC (Luo and Hu, 2023). Wang et al. reported that Huaier granule inhibited recurrence and metastasis after thermal ablation in patients with early-stage HCC (Wang et al., 2021). In this study, the OS rate of patients in the Huaier group was higher than that of the patients in the control group, regardless of whether the patient received resection, minimally invasive therapy, or palliative therapy. Moreover, few studies have explored the dose-response relationship between Huaier granules and OS. In this study, Huaier users exhibited significantly reduced mortality rate in a dose-response manner compared with that of non-Huaier users, and a significant effect of Huaier granule was observed on OS rate after adjusting for confounding factors. The aHR for Huaier users after PSM was 0.48, 0.23, 0.16 for 3–12, 12–24, and >24 months, respectively, suggesting that the patients treated with Huaier granule for longer duration had higher survival rate.

A previous study demonstrated that integrated Chinese and Western medicines can significantly reduce mortality risk in the subgroup of HCC patients with CTP class A (Sun et al., 2018). As shown in this study, the patients with different CTP classes benefited significantly from Huaier therapy, indicating that Huaier granule has therapeutic efficacy for patients with severe liver function. The efficacy of Huaier granules in patients with multiple clinical stages was also observed. Regardless of the tumor size (<5 or ≥5 cm), AFP level (<400 or ≥400 ng/mL), and BCLC stages (0–A, B, C–D), the 3-year OS rate of Huaier users was significantly higher than that of non-Huaier users. Furthermore, according to a forest plot, Huaier use was associated with lower mortality risk for most patient subgroups. Therefore, this cohort study provides evidence that Huaier granule, as an effective alternative therapeutic strategy, may improve the survival rate of patients with HCC. These results will be help clinicians in providing effective treatments and improving patient outcomes.

The effects and effectiveness of Huaier granule has been elucidated based on clinical, *in vitro*, and *in vivo* studies. Several studies have suggested that Huaier exerts antitumor effects by inhibiting the expression of yes-associated protein (YAP) and apoptosis-related proteins (Shan et al., 2017; Tao et al., 2018). Zhong et al. revealed that Huaier extract inhibits HepG2 cell growth in a dose-dependent manner (Zhong et al., 2018). Moreover, Huaier therapy is associated with the regulation of related pathways. Huaier granule has potential advantages in antitumor therapies involving multiple targets and pathways. n-Butanol extract of Huaier can inhibit the syntenin/STAT3 signaling pathway to inhibit tumor growth and metastasis (Shi et al., 2022). Huaier has been verified to have anti-cancer effects by inhibiting cancer cell growth and energy metabolism through the PI3K/AKT/HIF-1α pathway (Liu et al., 2021). Other studies have reported that Huaier granules regulate the proliferation, angiogenesis, and metastasis of HCC cells by the JNK, P38-MAPK, and YAP/CREB signaling pathways (Zhang et al., 2015; Zou et al., 2015; Niu et al., 2020).

This observational study elucidated the efficacy of Huaier granule in patients with HCC at different stages. Despite the significant findings of this study, it has certain limitations. First, although the baseline characteristics of the patients were adjusted according to PSM, several potential confounders may have been overlooked. Therefore, a large prospective clinical trial is required to confirm the clinical benefits of Huaier in patients with HCC. Second, this was a single-center cohort study and the sample size was small. The effect of Huaier on female and diabetes subgroups was not statistically significant; therefore, large multicenter, randomized controlled trials with large sample size are warranted. Finally, although Huaier use is related to lower 3-year mortality rate than that of non-Huaier users, it is necessary to further evaluate the effects of Huaier on long-term survival rate using a larger sample size.

## Conclusion

In the present study, Huaier granule treatment significantly increased OS rate in patients with HCC at different clinical stages; therefore, Huaier granule should be used in clinical practice for a favorable prognosis of HCC.

## Data Availability

The raw data supporting the conclusions of this article will be made available by the authors, without undue reservation.
